# ECDC’s latest publications

**DOI:** 10.2807/1560-7917.ES.2018.23.26.1806282

**Published:** 2018-06-28

**Authors:** Eurosurveillance editorial team

**Affiliations:** 1 European Centre for Disease Prevention and Control (ECDC), Stockholm, Sweden

**Keywords:** infectious diseases, public health, Europe

 

**Rapid risk and outbreak assessments**

 

Emergence of resistance to ceftazidime-avibactam in carbapenem-resistant Enterobacteriaceae

Date of publication: 13 June 2018

https://ecdc.europa.eu/en/publications-data/rapid-risk-assessment-emergence-resistance-ceftazidime-avibactam-carbapenem

 

Carbapenem-resistant Enterobacteriaceae - first update

Date of publication: 7 June 2018

https://ecdc.europa.eu/en/publications-data/rapid-risk-assessment-carbapenem-resistant-enterobacteriaceae-first-update

 

Mass gathering event, FIFA World Cup, Russia 2018

Date of publication: 28 May 2018

https://ecdc.europa.eu/en/publications-data/rapid-risk-assessment-mass-gathering-event-fifa-world-cup-russia-2018

 

Ebola virus disease outbreak in Equateur Province, Democratic Republic of the Congo

Date of publication: 25 May 2018

https://www.ecdc.europa.eu/en/publications-data/rapid-risk-assessment-ebola-virus-disease-outbreak-equateur-province-democratic-0

 

Multi-country outbreak of hepatitis A virus genotype IA infections affecting EU countries in 2018

Date of publication: 21 May 2018

https://www.ecdc.europa.eu/en/publications-data/rapid-risk-assessment-multi-country-outbreak-hepatitis-virus-genotype-ia

 

Extensively drug-resistant (XDR) *Neisseria gonorrhoeae* in the United Kingdom and Australia

Date of publication: 7 May 2018

https://ecdc.europa.eu/en/publications-data/rapid-risk-assessment-extensively-drug-resistant-xdr-neisseria-gonorrhoeae-united

 

Hospital-acquired malaria infections in the European Union

Date of publication: 30 April 2018

https://ecdc.europa.eu/en/publications-data/rapid-risk-assessment-hospital-acquired-malaria-infections-european-union

 

*Candida auris* in healthcare settings – Europe

Date of publication: 23 April 2018

https://ecdc.europa.eu/en/publications-data/rapid-risk-assessment-candida-auris-healthcare-settings-europe

 

Dengue outbreak in Réunion, France

Date of publication: 16 April 2018

https://ecdc.europa.eu/en/publications-data/rapid-risk-assessment-dengue-outbreak-reunion-france

 

**Scientific advice**

****

Public health guidance on active case finding of communicable diseases in prison settings

Date of publication: 23 May 2018

https://ecdc.europa.eu/en/publications-data/public-health-guidance-active-case-finding-communicable-diseases-prison-settings

****

**Technical reports**

 

Laboratory testing of non-partner sperm donors

Date of publication: 20 June 2018

https://ecdc.europa.eu/en/publications-data/laboratory-testing-non-partner-sperm-donors

 

HEPSA – health emergency preparedness self-assessment tool, User guide

Date of publication: 20 June 2018

https://ecdc.europa.eu/en/publications-data/hepsa-health-emergency-preparedness-self-assessment-tool-user-guide

 

Towards One Health preparedness

Date of publication: 24 May 2018

https://ecdc.europa.eu/en/publications-data/towards-one-health-preparedness

 

Social determinants and risk factors in tuberculosis surveillance in the EU/EEA

Date of publication: 24 May 2018

https://ecdc.europa.eu/en/publications-data/social-determinants-and-risk-factors-tuberculosis-surveillance-eueea

 

Molecular typing of *Neisseria gonorrhoeae* – a study of 2013 isolates

Date of publication: 16 May 2018

https://ecdc.europa.eu/en/publications-data/molecular-typing-neisseria-gonorrhoeae-study-2013-isolates

 

EU Laboratory Capability Monitoring System (EULabCap): Report on 2016 survey of EU/EEA country capabilities and capacities

Date of publication: 2 May

https://ecdc.europa.eu/en/publications-data/eu-laboratory-capability-monitoring-system-eulabcap-report-2016-survey-eueea

 

**Surveillance reports**

 

Monthly measles and rubella monitoring report, June 2018

Date of publication: 8 June 2018

https://ecdc.europa.eu/en/publications-data/monthly-measles-and-rubella-monitoring-report-june-2018

 

Influenza virus characterisation, Summary Europe, March 2018

Date of publication: 4 June 2018

https://ecdc.europa.eu/en/publications-data/influenza-virus-characterisation-summary-europe-march-2018

 

Surveillance of antimicrobial consumption in Europe 2013-2014

Date of publication: 28 May 2018

https://ecdc.europa.eu/en/publications-data/surveillance-antimicrobial-consumption-europe-2013-2014

 

Monthly measles and rubella monitoring report, May 2018 

Date of publication: 18 May 2018

https://ecdc.europa.eu/en/publications-data/monthly-measles-and-rubella-monitoring-report-may-2018

 

Incidence and attributable mortality of healthcare-associated infections in intensive care units in Europe, 2008-2012 

Date of publication: 3 May 2018

https://ecdc.europa.eu/en/publications-data/incidence-and-attributable-mortality-healthcare-associated-infections-intensive

 

Measles and rubella surveillance – 2017

Date of publication: 23 April 2018

https://ecdc.europa.eu/en/publications-data/annual-measles-and-rubella-monitoring-report-2017

 

Monthly measles and rubella monitoring report, April 2018

Date of publication: 13 April 2018

https://ecdc.europa.eu/en/publications-data/monthly-measles-and-rubella-monitoring-report-april-2018

 

**Annual Epidemiological Report series on communicable diseases in Europe**

****

*Clostridium difficile* infections - Annual Epidemiological Report for 2016

Date of publication: 20 June 2018

https://ecdc.europa.eu/en/publications-data/clostridium-difficile-infections-annual-epidemiological-report-2016

****

Cholera - Annual Epidemiological Report for 2015

Date of publication: 14 June 2018

https://ecdc.europa.eu/en/publications-data/cholera-annual-epidemiological-report-2015

****

Hepatitis A - Annual Epidemiological Report for 2015

Date of publication: 14 June 2018

https://ecdc.europa.eu/en/publications-data/hepatitis-annual-epidemiological-report-2015

****

Antimicrobial consumption - Annual Epidemiological Report for 2016

Date of publication: 13 June 2018

https://ecdc.europa.eu/en/publications-data/antimicrobial-consumption-annual-epidemiological-report-2016

****

Antimicrobial consumption - Annual Epidemiological Report for 2015

Date of publication: 12 June 2018

https://ecdc.europa.eu/en/publications-data/antimicrobial-consumption-annual-epidemiological-report-2015

 

Antimicrobial consumption - Annual Epidemiological Report for 2014

Date of publication: 11 June 2018

https://ecdc.europa.eu/en/publications-data/antimicrobial-consumption-annual-epidemiological-report-2014

 

Healthcare-associated infections acquired in intensive care units - Annual Epidemiological Report for 2016

Date of publication: 4 May 2018

https://ecdc.europa.eu/en/publications-data/healthcare-associated-infections-acquired-intensive-care-units-annual-0

 

Surgical site infections - Annual Epidemiological Report for 2016

Date of publication: 4 May 2018

https://ecdc.europa.eu/en/publications-data/surgical-site-infections-annual-epidemiological-report-2016

 

Botulism - Annual Epidemiological Report for 2015

Date of publication: 30 April 2018

https://ecdc.europa.eu/en/publications-data/botulism-annual-epidemiological-report-2015

 

Campylobacteriosis - Annual Epidemiological Report for 2015

Date of publication: 30 April 2018

https://ecdc.europa.eu/en/publications-data/campylobacteriosis-annual-epidemiological-report-2015

 

Brucellosis - Annual Epidemiological Report for 2015

Date of publication: 30 April 2018

https://ecdc.europa.eu/en/publications-data/brucellosis-annual-epidemiological-report-2015

 

Giardiasis - Annual Epidemiological Report for 2015

Date of publication: 30 April 2018

https://ecdc.europa.eu/en/publications-data/giardiasis-annual-epidemiological-report-2015

 

Leptospirosis - Annual Epidemiological Report for 2015

Date of publication: 30 April 2018

https://ecdc.europa.eu/en/publications-data/leptospirosis-annual-epidemiological-report-2015

 

Listeriosis - Annual Epidemiological Report for 2015

Date of publication: 30 April 2018

https://ecdc.europa.eu/en/publications-data/listeriosis-annual-epidemiological-report-2015

 

Salmonellosis - Annual Epidemiological Report for 2015

Date of publication: 30 April 2018

https://ecdc.europa.eu/en/publications-data/salmonellosis-annual-epidemiological-report-2015

 

Shigellosis - Annual Epidemiological Report for 2015

Date of publication: 30 April 2018

https://ecdc.europa.eu/en/publications-data/shigellosis-annual-epidemiological-report-2015

 

Yersiniosis - Annual Epidemiological Report for 2015

Date of publication: 30 April 2018

https://ecdc.europa.eu/en/publications-data/yersiniosis-annual-epidemiological-report-2015

 

Typhoid and paratyphoid fever - Annual Epidemiological Report for 2015

Date of publication: 30 April 2018

https://ecdc.europa.eu/en/publications-data/typhoid-and-paratyphoid-fever-annual-epidemiological-report-2015

 

Anthrax - Annual Epidemiological Report for 2015

Date of publication: 27 April 2018

https://ecdc.europa.eu/en/publications-data/anthrax-annual-epidemiological-report-2015

 

**Mission reports**

ECDC country visit to Romania to discuss antimicrobial resistance issues

Date of publication: 21 June 2018

https://ecdc.europa.eu/en/publications-data/ecdc-country-visit-romania-discuss-antimicrobial-resistance-issues

****

**Corporate publications**

****

ECDC public health microbiology strategy 2018–2022

Date of publication: 20 June 2018

https://ecdc.europa.eu/en/publications-data/ecdc-public-health-microbiology-strategy-2018-2022

****

**ECDC communicable disease threats report**

Date of publication: every Friday

https://ecdc.europa.eu/en/threats-and-outbreaks/reports-and-data/weekly-threats

**

**Scientific peer-reviewed publications**

Publications by ECDC staff in scientific journals

Updated monthly

https://ecdc.europa.eu/en/publications-data?f%5b0%5d=output_types%3A1247

